# False Impressions

**Published:** 1888-11-24

**Authors:** Caroline Fothergill

**Affiliations:** Author of "An Enthusiast," "A Voice in the Wilderness," etc.


### FALSE IMPRESSIONS.

By Caroline Fothergill, Author of "An Enthusiast," "A Voice in the Wilderness," etc.

*(Continued from p. 111.)*

### Chapter VIII.—Jack.

Beatrice continued her readings all through the winter. They had become very popular. Most of the children came now because they were interested in what they heard, and numbers of them came so regularly that Beatrice knew them quite well, both by name and face, and had many chats with them. One boy in particular she liked, a bright-faced little lad about eight years old, Jack Wilson by name. He was a handsome little fellow, with beautiful brown eyes and a mass of curly brown hair. He was a very regular attendant, and always sat in the front row of chairs, his hands in his pockets, drinking in all he heard. He had a particular love for fairy tales, and it was so natural and healthy a love in a child of his age, and argued a nature as yet unspoiled by his surroundings, that Beatrice often found herself humouring it by reading fairy tales when other boys were clamouring for adventures and crocodiles—the favourite beast of their imagination. He came so regularly that she experienced a feeling of disappointment when one evening he was absent from the reading. She thought he might have been kept at home for some reason, and made no remark on his absence; but on the following Thursday he again did not come. Still she said nothing. She was chary of such inquiries: so many and often such odd reasons kept the children at home. But when on the third Thursday he still failed to appear, she felt uneasy, and asked another boy, who was rather a friend of Jack's, if he knew why he had ceased coming?

"Ay," said the boy, "he did;" and then it came out that Jack had been kept at home by typhoid fever, but he had not had it very badly, and now he was getting better, he wanted sadly to come again. This boy had seen him only this evening, and Jack had blubbered awful at the disappointment of missing the reading. The lad further added that he had promised, when the stories were over, to go in and see Jack, and repeat to him to the best of his ability, what he had heard this evening.

Beatrice pondered. An idea had flashed into her mind, and she was strongly tempted to carry it out. It was still early; she had begun rather before time, and the reading had not been a long one. Suppose she were to go and visit her little sick friend, see how he was, and if she found him well enough, read over again to him some of this evening's stories, instead of leaving them to be mangled by the lad at her side. Laura of course would disapprove of her venturing into what she would call "a den of pestilence," but Laura would know nothing about it until it was over, if indeed she ever told her of it. Her clothes could be destroyed, and for herself she had no fear at all. So after a moment's hesitation, she turned to the boy and asked :—

"Where does Jack Wilson live? Is it far from here?"

The boy had been watching her face. Perhaps he guessed her purpose, for he said eagerly—

"No, missis; it's close to—not above five minutes."

"Then I'll tell you what," she said, rising; "you take me to Jack Wilson's house, and if he's awake and well enough to listen, I will read him what I have been reading to you this evening."

The lad expressed his delight in characteristic fashion, a little toned down by the company in which he found himself, and they at once set off to Jack's home. It was a frosty, moonlight night, and Beatrice could see that the street, when they reached it, was a very decent one, as such streets go. As they walked along it her guide vouchsafed the cheering information—

"There's a deal of fever here; two died last week; but Wilson's not been so bad."

They reached the house, knocked, and were admitted by a very tidy-looking young woman, to whom Beatrice introduced herself, and explained her errand, adding that if Jack were well enough, and she would be putting no one to inconvenience, she would like to read him the stories which the other children had heard.

The working-people of Mayfield are slow of belief, and it is possible her story might not have gained credence had it not been for the presence of her guide, who was well known to the woman, and who emphatically endorsed every word she said. The woman gave her a long look, and then admitted her, neither willingly nor unwillingly as it seemed, rather as though impelled by Beatrice's manner she could not do otherwise.

Jack was in bed upstairs, in a room which was clean enough, but rather stuffy, and Beatrice at once realised that if she ran any risk it would be here, sitting in this atmosphere, and breathing this air, as she read aloud to the child. Even had she had any wish to draw back, however, it was too late to do it now, and the little lad welcomed her so heartily in his weak little voice that she could not help feeling glad she had come, and after talking to him for a little while, she began to read, and read in her soft clear voice until Jack lay asleep, with his thin hand in hers.

As soon as she saw that the child was asleep, she ceased reading, and would have gone away, but he held her hand firmly, and she could not withdraw it without waking him. At last, he himself, in the restlessness of his sleep, let it go, and after looking at him for a minute or two, she got up and noiselessly left the room. The door had been closed, and it was not until she was halfway down the stairs leading into the kitchen that she heard a voice speaking which certainly belonged to no Mayfield workman, and which in a minute she recognised as George Murray's.

She stood still in dismay. What should she do? How avoid this meeting? An instant's reflection showed her that it could not be avoided; they must have heard her open the door upstairs, and her descending footsteps. After an almost imperceptible pause, she continued her way downstairs, and, in coming into the kitchen, found herself face to face with Murray.

She would have passed him with a bow and gone out, after saying a few words to the woman of the house; but he frustrated her intention by coming suddenly forward and placing himself between her and the door.

"Good evening, Miss Stanford," he began; "who would have thought of seeing you here?"

"Or you!" she answered quietly.

"Oh, I—I come here often. Mrs. Wilson knows how frequent a visitor I am; and I have been coming very often lately to see how that little lad upstairs is getting on. But you, I cannot, in my imagination, associate you with a place of this kind."

"Why should not I come to see Jack as well as you? Had I known of his illness I should have come before. I only heard of it to-night by accident."

"But do you know what his illness has been? Are you aware of the risk you run?"

"I suppose there is a risk, but I do not think I shall take any harm. I am naturally strong and healthy."

He was looking at her fixedly with his keen, deep eyes, and at her last words he half smiled, as he said—

"And also naturally very brave, or very foolhardy—which is it?"

She drew herself up a little, as she answered—

"I cannot help you to decide that point." Then, after a few words to the woman of the house, who was now loud in her thanks for her kindness in coming, she said a general "Good evening," and would again have gone out, but again George intercepted her.

"Allow me to see you to the tram," he said hastily.

"Thank you," she said. "I can go alone."

"Excuse me," he persisted, "I cannot permit it."

She turned upon him with a sudden flash.

"I beg of you to stay where you are," she said almost doggedly; and he could only fall back and let her go.

She bit her lips as she walked along. "Brave or foolhardy?" he would allow her nothing. He had been very hard. Why should she care? But she did care. She cared so much that her throat ached with the effort she made to repress her tears as she walked along. She had been so happy in doing this little kindness, now it was spoiled, and she was called a fool for her pains. Her spirit rose against it for a moment, but it was useless trying to conceal that she felt more miserable than angry. She was deeply hurt, for by some means George had impressed her as no one else had ever done. She found herself thinking of him and desiring his good opinion, in a way which was unaccountable even to herself, and which at times caused her some uneasiness.

Although this was the first time she had met George during her visits, it was by no means her first going amongst the working-people. She had gone amongst them a good deal, paying friendly calls, generally in the evening when the day's work was done, and people had time to sit and talk. She had by this time many friends amongst them, and always enjoyed the intercourse she had with them and their children. They frequently spoke of what was being done by the Murray Society, and it was inevitable that George's name should be constantly mentioned. Beatrice seldom spoke of him, but she never checked her friends when they did, and by degrees she found herself becoming possessed of a portrait so different from the original as she had imagined it, that sometimes she wondered if there were two George Murray's—one known to her and one to these people. She came to the conclusion, with rather a sarcastic smile, that probably there were.

She had once, in speaking of him to Laura, called him an "odious man," and though she no longer thought him odious, she had believed that he was habitually caustic and cynical. It appeared now, however, that he was cheerful, large-hearted, and generous. These people were constantly singing his praises. He seemed to be the good genius of the district. He had given this man help in direst need; he had rescued that man's tools from the pawnshop, and set him on his feet again; he had befriended the cripple in another family, so that the lad, instead of begging was now earning a decent livelihood at a trade for which he had a natural taste. It would seem that he seldom gave direct money help, his opinion—probably the result of considerable experience—being that few people are really benefited in that way. But he was always ready and willing to help them to increase their earnings, or to show them how to make the most of what they already had.

"And," continued the woman who was telling Beatrice all this, "he has such a pleasant way wi' him, for all he's such a grand gentleman—and anyone can see he is; unless you've done wrong, you're never afraid to look him in th' face. And his smile is that pleasant it puts new heart into you, however down you may be. Eh, but there's not many like Mr. Murray; is there, Jim?"

Jim responded to this appeal by emphatically endorsing his wife's testimony, adding on his own account:

"Ay, it's a pleasure to see Mr. Murray, if all's square; but if it isn't I'd rather meet the devil hissel', s'help me! I've seen chaps, Miss Stanford, as had told a lie, an' Mr. Murray had found it out, and then didn't he give it 'em! Oh, Lord! I wouldn't have been in their shoes. Not as he ever said much, he's not a talker, isn't Mr. Murray, but he'd say a few words an' just look at 'em, an' they shrivelled up like bits o' paper on top o' t' fire. There's no one like him for getting under a man's skin an' making it sting."

Beatrice heard this with mingled feeling, and Jim continued:—

"If there's one thing as Mr. Murray hates above another, it's a lie. He says a liar's the meanest trade going, an' one no man as is a man would ever take up; an' he's just as hard, or harder, on th' women. I've seen him make a woman i' this street wince as ne'er winced i' her life afore, not for nobody."

So it was always. Go where she would, she heard always the same—that he was wise, kind, firm, and upright, just to man and beast, loved deeply by all who knew him; feared, too, by a few.

All these things she kept to herself; she shared none with Laura, and she went over them again and again, feeling that to this man she could do homage with the humblest Mayfield artisan. But he would not let her, he would not have her homage. Kind and friendly to the poorest of the poor, to her, the wealthy and beautiful woman in his own rank, he was coldly polite, indifferent, and careless; and she, by the mere force of his will, was forced to accept the situation, however much she wished to change it. It was not she who kept him at arm's length, but he who held her in that uncomfortable and isolated position. It was a humiliating situation, and she chafed against it, but in vain.

George's feelings for her had undergone a change too. He had never again written about her to Dr. Temple, and he had long ago given up his belief that she had been guilty of any departure from the truth. He heard as much of her from the working people as she did of him, and what he heard was equally to her credit, so that he, knowing the caution of these people, and aware of the probation all candidates for their favour must go through, knew that she must have amply proved her right to the judgments which were passed upon her. Insensibly she was becoming more and more to him, and as he, misled by her manner, which was only the outcome of his to her, was convinced that he was not advancing in her regard, and being by nature slow to accept his wishes as facts, he curbed his impulses and toned down his manner to a degree of which he was himself not aware. He would not yield to the growing attraction he felt in herself and her society; he set himself against it, and encouraged all his former feelings in her disfavour. Then came this little incident in the Wilsons' house, from which he went home in a rage with himself, knowing he had gone too far, and yet ignorant how he could put matters right, even though he longed to do so. At last he decided to leave things to chance. So we see them at the end of the winter, both anxious for a nearer acquaintance, both ready to take advantage of any accident which might bring it about. Nor, considering the slowness with which human affairs move, was the opportunity long in coming.

(*To be continued.*)

